# Early increase of the synaptic blood marker β‐synuclein in asymptomatic autosomal dominant Alzheimer's disease

**DOI:** 10.1002/alz.70146

**Published:** 2025-04-10

**Authors:** Patrick Oeckl, Benjamin Mayer, Randall J. Bateman, Gregory S. Day, Nick C. Fox, Edward D. Huey, Laura Ibanez, Takeshi Ikeuchi, Mathias Jucker, Jae‐Hong Lee, Johannes Levin, Jorge J. Llibre‐Guerra, Francisco Lopera, Eric McDade, John C. Morris, Yoshiki Niimi, Jee Hoon Roh, Raquel Sánchez‐Valle, Peter R. Schofield, Markus Otto, the Dominantly Inherited Alzheimer Network

**Affiliations:** ^1^ Department of Neurology Ulm University Hospital Ulm Germany; ^2^ German Center for Neurodegenerative Diseases (DZNE) Ulm Ulm Germany; ^3^ Institute for Epidemiology and Medical Biometry University of Ulm Ulm Germany; ^4^ Department of Neurology Washington University School of Medicine Saint Louis Missouri USA; ^5^ Department of Neurology Mayo Clinic in Florida Jacksonville Florida USA; ^6^ The Dementia Research Centre Department of Neurodegenerative Disease UCL Queen Square Institute of Neurology London UK; ^7^ Department of Psychiatry and Human Behavior Alpert Medical School of Brown University Providence Rhode Island USA; ^8^ Department of Psychiatry Department of Neurology and NeuroGenomics and Informatics Center Washington University Saint Louis Missouri USA; ^9^ Brain Research Institute Niigata University Niigata Japan; ^10^ German Center for Neurodegenerative Diseases (DZNE) Tübingen Tübingen Germany; ^11^ Hertie‐Institute for Clinical Brain Research University of Tübingen Tübingen Germany; ^12^ Department of Neurology Asan Medical Center University of Ulsan College of Medicine Seoul South Korea; ^13^ Department of Neurology LMU University Hospital LMU Munich Munich Germany; ^14^ German Center for Neurodegenerative Diseases (DZNE) Munich Munich Germany; ^15^ Munich Cluster for Systems Neurology (SyNergy) Munich Germany; ^16^ Neuroscience Group of Antioquia (GNA) Medicine Faculty Antioquia University Medellín Colombia; ^17^ Department of Neurology Washington University School of Medicine Saint Louis Missouri USA; ^18^ Department of Neurology and the Knight Alzheimer Disease Research Center Washington University Saint Louis Missouri USA; ^19^ Unit for Early and Exploratory Clinical Development The University of Tokyo Tokyo Japan; ^20^ Departments of Neurology and Physiology Korea University Anam Hospital Korea University College of Medicine Seoul South Korea; ^21^ Alzheimer's Disease and Other Cognitive Disorders Unit. Hospital Clínic de Barcelona FRCB‐IDIBAPS University of Barcelona Barcelona Spain; ^22^ Neuroscience Research Australia Sydney New South Wales Australia; ^23^ School of Biomedical Sciences University of New South Wales Sydney New South Wales Australia; ^24^ Department of Neurology Martin‐Luther‐University Halle‐Wittenberg Halle (Saale) Germany

**Keywords:** asymptomatic mutation carriers, autosomal dominant Alzheimer´s disease, β‐synuclein, blood biomarker, preclinical Alzheimer´s disease, synaptic degeneration

## Abstract

**INTRODUCTION:**

β‐synuclein is a promising blood marker to track synaptic degeneration in Alzheimer's disease (AD) but changes in preclinical AD are unclear.

**METHODS:**

We investigated serum β‐synuclein in 69 cognitively unimpaired mutation non‐carriers, 78 cognitively unimpaired AD mutation carriers (asymptomatic AD), and 31 symptomatic mutation carriers from the Dominantly Inherited Alzheimer Network.

**RESULTS:**

β‐synuclein levels were already higher in asymptomatic AD mutation carriers compared to non‐carriers and highest in symptomatic carriers. Longitudinal trajectories and correlation analyses indicated that β‐synuclein levels start to rise after amyloid deposition preceding axonal degeneration, brain atrophy and hypometabolism, and cognitive decline. β‐synuclein levels were associated with cognitive impairment and gradually increased with declining cognition.

**DISCUSSION:**

Our study supports the use of blood β‐synuclein to track synaptic changes in preclinical AD and as a surrogate marker for cognitive impairment which might be used in early diagnosis and to support patient selection and monitoring of treatment effects in clinical trials.

**Highlights:**

- Blood β‐synuclein levels were already higher in asymptomatic Alzheimer's disease (AD) mutation carriers.
- Blood β‐synuclein levels were highest in symptomatic AD mutation carriers.
- Blood β‐synuclein levels start to rise 11 years before symptom onset.
- Rise of β‐synuclein precedes axonal degeneration, brain atrophy, and cognitive decline.
- β‐synuclein levels gradually increased with declining cognition.

## 1 BACKGROUND

Synaptic degeneration is a major hallmark of Alzheimer's disease (AD) and a pathological correlate of memory impairment. Monitoring of synaptic changes in AD patients is therefore of importance for diagnosis, prognosis, and assessing disease progression. Furthermore, an essential aim in the development of disease‐modifying therapies for AD is to slow or stop synaptic degeneration. Biomarkers provide an important tool to track beneficial effects on synaptic loss in clinical trials. Due to the strong association of synaptic loss with memory impairment,^1^ synaptic markers may also serve as surrogate markers for cognitive decline and could provide objective measures of therapeutic benefit and potentially earlier than neuropsychological tests allowing clinical trials to be conducted faster, with fewer participants, and at lower costs

Several synaptic markers have been extensively investigated in the cerebrospinal fluid (CSF) in symptomatic AD patients, including β‐synuclein, neurogranin, and synaptosomal‐associated protein, 25kDA.^2^ Indeed, recent trials of anti‐amyloid drugs that showed cognitive benefit also showed that synaptic markers in CSF improved upon treatment^3^ thereby providing proof‐of‐concept for the use of synaptic markers in body fluids in AD trials. However, less is known about synaptic degeneration in the preclinical phase of AD. This is due to the current reliance on symptomatic diagnosis of AD making this phase less accessible for clinical research. In addition, CSF samples are more difficult to collect from preclinical AD patients and are often not available from population‐based cohort studies. The characterization of synaptic degeneration in the preclinical phase of AD is of great importance to determine its position in the sequence of pathophysiological changes such as amyloid and tau deposition, brain atrophy, and cognitive impairment. This would also help assess how it might be used in (early) diagnosis, progression, and decision making for treatment or drug monitoring.

An easily accessible synaptic marker in blood could facilitate the characterization of synaptic changes in AD and advance the field similar to the implementation of other blood biomarkers such as neurofilaments or phosphorylated tau (p‐tau) species. Blood is easier and more convenient to collect for patients enabling tighter monitoring of synaptic degeneration. It is also advantageous for early diagnosis or stratification of prospective clinical trial participants and to study synaptic degeneration in large, population‐based cohorts in which CSF is often not available. Recently, we generated strong evidence that measurement of β‐synuclein, a presynaptic protein with highly specific expression in the brain, is a promising synaptic blood biomarker filling this important gap. In several independent studies, we showed higher β‐synuclein levels in the blood of sporadic AD cases, a significant correlation with cognitive decline, brain atrophy, amyloid, and tau pathology.^4^, ^5^, ^6^, ^7^ Furthermore, there was evidence that β‐synuclein in the blood is already raised in preclinical AD^5^ but as this was from cross‐sectional studies it was unclear at what time in the course of AD it starts to increase and how it relates to the trajectories of other mechanisms.

Asymptomatic autosomal dominant AD (ADAD) mutation carriers present a unique opportunity to study the preclinical phase of AD and to rank different mechanisms in the course of early AD pathophysiology.^8^ The Dominantly Inherited Alzheimer Network (DIAN) observational study is a worldwide multicenter initiative to longitudinally study dominantly inherited AD^9^ and substantially contributed to our understanding of the trajectories of many fluid and other biomarkers in the preclinical disease phase of AD. It provides a unique opportunity to study blood β‐synuclein levels in very early AD and assess the timing and relation of β‐synuclein changes to other pathological alterations.

The aim of the present study was to investigate serum β‐synuclein levels in asymptomatic AD mutation carriers (aMC) from the DIAN observational study compared to symptomatic mutation carriers (sMC) and mutation non‐carriers (NC) to characterize synaptic degeneration in the asymptomatic disease phase and the longitudinal trajectories compared to amyloid deposition ([^11^C]‐Pittsburgh compound B positron emission tomography [PiB PET]), CSF biomarkers (amyloid beta 42/40 ratio [Aβ42/40], total tau protein (t‐tau), p‐tau variants), axonal degeneration (serum neurofilament light chain [NfL]), brain atrophy (magnetic resonance imaging [MRI]) and metabolism ([^18^F]‐fluorodeoxyglucose [FDG] PET), and cognitive impairment (Mini‐Mental State Examination [MMSE], Clinical Dementia Rating Sum of Boxes [CDR‐SB], DIAN cognitive composite). These findings inform the ranking of synaptic degeneration in the sequence of pathophysiological mechanisms in ADAD and provide important information about the possible applications of a β‐synuclein blood test for diagnosis, prognosis, progression, and monitoring.

## 2 METHODS

### 2.1 Participants

We included samples from 182 participants of the DIAN observational study, which enrolls adult children of clinically affected ADAD mutation carriers that have a 50% risk of inheriting an ADAD mutation (participants recruited between 2009 and 2015, DIAN request T2204). The design of the DIAN study has been described previously (ClinicalTrials.gov number NCT00869817).^9^, ^10^ Four NCs were excluded because of the diagnosis of possible AD (*n* = 1) or a global CDR > 0 (*n* = 3). The final cohort thus consisted of 178 participants including 69 NCs, that is, cognitively unimpaired subjects without a known AD mutation and a global CDR = 0; 78 aMC, that is, cognitively unimpaired subjects carrying an ADAD mutation and with a global CDR = 0; and 31 sMC, that is, cognitively impaired ADAD mutation carriers with a global CDR > 0. All participants gave written informed consent and all procedures were approved by the institutional review board (IRB) of Washington University (Saint Louis, MO, USA) and the local IRBs of the participating sites. Characteristics of participants are described in Table 1 and are based on data freeze 15 from the DIAN study.

**RESEARCH IN CONTEXT**

1. **Systematic review**: We reviewed the literature in PubMed and previous studies support higher blood β‐synuclein levels in preclinical Alzheimer's disease (AD). However, changes of blood β‐synuclein in AD mutation carriers have not been investigated before and the dynamics in the asymptomatic phase of AD and the temporal relation to other pathophysiological mechanisms is unclear.
2. **Interpretation**: The study showed that serum β‐synuclein levels were already higher in asymptomatic AD. It indicated that levels became abnormal ≈ 11 years before symptom onset and represented an early marker of AD pathophysiology preceding changes in brain structure and metabolism, cognition, and axonal neurodegeneration.
3. **Future directions**: Our study supports the use of blood β‐synuclein to track synaptic changes in preclinical AD and as a surrogate marker for cognitive impairment. Future studies must evaluate how it can contribute to early diagnosis and whether it can support patient selection and monitoring of treatment effects in clinical trials. Longitudinal studies are needed to confirm our temporal estimates and determine that findings in asymptomatic autosomal dominant AD are generalizable to sporadic AD.

**TABLE 1:** Demographic and clinical characteristics of participants at baseline.

	*N*	Mutation non‐carrier (NC)	Mutation carrier asymptomatic (aMC)	Mutation carrier symptomatic (sMC)	*p* value
*N* (% female)	178	69 (65%)	78 (62%)	31 (52%)	0.16
Age (years)	178	38.4 (32.3–47)	35.4 (30.2–41.8)	46.6 (42.1–52.6)^a^, ^b^	< 0.0001
*APOE* ε4 positive (%)	178	33	32	39	0.8
Education (years)	178	15 (13–16)	14.5 (12–17)	12 (11–14)^c^	0.0007
CDR‐SB	178	0 (0–0)	0 (0–0)	5.5 (5–7)^b^, ^d^	< 0.0001
MMSE	177 (69,78,30)	30 (29–30)	29 (28–30)	18 (12–21)^b^, ^d^	< 0.0001
DIAN cognitive composite (*z* score)	177 (69,78,30)	0.242 (0.07–0.449)	0.195 (−0.065–0.46)	−1.173 (−1.534–0.834)^b^, ^d^	< 0.0001
Serum β‐synuclein (pg/mL)	178	5.25 (4.63–6.66)	7.29 (5.89–8.59)^e^	14.35 (11.7–18.4)^b^, ^d^	< 0.0001
Serum NfL (pg/mL)	169 (65,75,29)	18.8 (13.6–27.6)	20.2 (16.4–30.7)	73.4 (61.9–88.5)^b^, ^d^	< 0.0001
CSF Aβ40 (pg/mL)	178	8431 (6831–10217)	8627 (6483–11113)	7487 (5967–9131)	0.18
CSF Aβ42 (pg/mL)	178	777 (585–975)	554 (370–782)^d^	326 (274–375)^d^, ^f^	< 0.0001
CSF tTau (pg/mL)	178	212 (180–260)	287 (220–520)^g^	833 (586–1200)^b^, ^d^	< 0.0001
CSF pTau181 (pg/mL)	178	25.1 (19.5–29.9)	36.3 (26.1–67.6)^h^	139.4 (105.7–190.9)^b^, ^d^	< 0.0001
CSF Aβ42/40 ratio	178	0.0924 (0.0878–0.0966)	0.0629 (0.0515–0.0957)^d^	0.0437 (0.0337–0.0598)^d^, ^i^	< 0.0001
CSF pT181/T181	147 (57,65,25)	21.4 (20.2–22.3)	24.4 (22.1–29.6)^d^	35.6 (31.9–41.6)^d^, ^i^	< 0.0001
CSF pT205/T205	147 (57,65,25)	0.336 (0.271–0.371)	0.42 (0.34–0.576)^h^	1.14 (1.006–1.271)^b^, ^d^	< 0.0001
CSF pT202/T202	147 (57,65,25)	3.2 (2.54–3.55)	2.83 (2.12–3.62)	2.64 (2.01–2.99)^j^	0.05
CSF pT217/T217	146 (57,64,25)	1.11 (0.96–1.28)	2.4 (1.28–4.95)^d^	9.6 (7.81–10.95)^b^, ^d^	< 0.0001
Cortical PiB Pet SUVR	147 (60,66,21)	1.03 (0.99–1.08)	1.47 (1.11–2.38)^d^	2.81 (2.15–4.59)^d^, ^i^	< 0.0001
Precuneus FDG PET SUVR	154 (60,71,23)	1.87 (1.79–1.96)	1.88 (1.78–2)	1.49 (1.35–1.61)^b^, ^d^	< 0.0001
Cortical FDG PET SUVR	154 (60,71,23)	1.69 (1.61–1.76)	1.71 (1.6–1.82)	1.41 (1.36–1.51)^b^, ^d^	< 0.0001
Precuneus thickness (mm)	165 (66,74,25)	4.77 (4.6–4.95)	4.74 (4.57–4.93)	3.71 (3.53–4)^b^, ^d^	< 0.0001
Hippocampal volume (mm^3^)	165 (66,74,25)	8769 (8089–9362)	8808 (8095–9256)	6651 (5535–7386)^b^, ^d^	< 0.0001

*Note*: Data are given as median and interquartile ranges. Sex distribution and *APOE* ε4 positivity were compared with the chi‐square test; age, education, and clinical scores with Kruskal–Wallis and Dunn post hoc test; fluid and imaging biomarkers with multivariate general linear regression (log10‐transformed if not normally distributed) including age, sex, education, and *APOE* ε4 status as covariates and Sidak adjustment for multiple comparisons.

Abbreviations: Aβ, amyloid beta; *APOE*, apolipoprotein E; CDR‐SB, Clinical Dementia Rating Sum of Boxes; CSF, cerebrospinal fluid; DIAN, Dominantly Inherited Alzheimer's Network; FDG, fluorodeoxyglucose; MMSE, Mini‐Mental State Examination; NfL, neurofilament light chain; PET, positron emission tomography; PiB, Pittsburgh compound B; pT, phosphorylated tau; SUVR, standardized uptake value ratio.

^a^ *p* = 0.001 versus NC

^b^ *p *< 0.0001 versus aMC

^c^ *p* = 0.0007 versus NC, *p* = 0.0027 versus aMC

^d^ *p *< 0.0001 versus NC

^e^ *p* = 0.001 versus NC

^f^ *p* = 0.011 versus aMC

^g^ *p *< 0.01 versus NC

^h^ *p *< 0.001 versus NC

^i^ *p *< 0.001 versus aMC

^j^ *p *< 0.05 versus NC

Distribution of mutations was as follows: *PSEN1* (aMC *n* = 58, sMC *n* = 28), *PSEN2* (aMC *n* = 9), *APP* (aMC *n* = 11, sMC *n* = 3). The presence or absence of an ADAD mutation was confirmed using polymerase chain reaction–based amplification of the relevant exon, followed by Sanger sequencing. At least one follow‐up visit was available for 49 participants (NC *n* = 16, aMC *n* = 20, sMC *n* = 13) including two subjects from the aMC group that converted to the symptomatic stage during follow‐up. Follow‐up visits in DIAN are performed in intervals of 1 to 3 years.

The estimated years to symptom onset (EYO) were calculated for both MC and NC. For asymptomatic individuals and NC in DIAN, EYO is based on the difference between age at visit and the mean age of symptom onset for this specific mutation or the parental age of onset if no mutation age is available (in NCs, the mutation of the affected family member). For sMC, the DIAN EYO is calculated using the difference between the age at visit and age at appearance of first symptoms.

### 2.2 Clinical assessment and sample collection

Participants underwent a comprehensive clinical and neuropsychological evaluation^10^ conducted by clinicians who were blinded to the mutation status of participants and to prior evaluations. The global CDR was used to define asymptomatic (global CDR = 0) and symptomatic AD (global CDR > 0). Further tests of cognitive performance that are reported in our study are the MMSE, CDR‐SB, and the DIAN cognitive composite, which is calculated as the mean of the *z* scores from four neuropsychological tests: MMSE, logical memory delayed recall (MEMUNITS), digit‐symbol substitution (WAIS), and animal naming (ANIMALS). The annual rate of change of the MMSE and CDR‐SB was calculated from the difference between the baseline and first available follow‐up visit divided by the time (in years) between these visits. CSF and blood were collected in the morning under fasting conditions by means of lumbar puncture and venipuncture.^10^

### 2.3 Determination of serum β‐synuclein by IP‐MS

Serum β‐synuclein was measured by immunoprecipitation mass spectrometry (IP‐MS) as previously described.^7^ In brief, 490µL aliquots of serum were mixed with an internal standard solution containing recombinant ^15^N‐β‐synuclein (rPeptide) and immunoprecipitated using magnetic beads coupled with an anti‐β‐synuclein antibody (EP1537Y from Abcam). Beads were washed with 50mM triethylammonium bicarbonate/0.1% *n*‐Dodecyl‐β‐D‐maltoside using a Kingfisher Apex instrument and eluted. β‐synuclein was digested by trypsin/LysC (Promega), and two proteotypic peptides were quantified by liquid chromatography multiple reaction monitoring (MRM; aa46–58 and aa61–85) using an Eksigent MicroLC200, Agilent 1260 pump, and Sciex QTRAP6500 mass spectrometer in MRM mode. Calibrators were prepared using recombinant human full‐length β‐synuclein (without tags) from rPeptide and the exact concentration of the β‐synuclein stock solution was quantified by amino acid analysis (Alphalyse A/S). Calibration range was 2 to 30 pg/mL. Serum quality control (QC) samples (low, medium, high) were included in all four runs (intraassay coefficient of variation [CV] 0.5%–9.5%, interassay CV 2.7%–7.7%). Samples were randomly assigned to the different runs and the analysts were blinded to participant data.

### 2.4 Determination of CSF biomarkers and serum NfL

CSF AD core biomarkers (Aβ40, Aβ42, t‐tau, p‐tau181) were measured by Lumipulse (Fujirebio) following standard procedures. CSF ratios of tau phosphorylation sites (pT181/T181, pT217/T217, pT205/T205, and pT202/T202) were measured by IP‐MS as previously described.^8^ Serum NfL was determined using Simoa (Quanterix).^11^

### 2.5 Imaging

Imaging data available in the DIAN study were recently described.^12^ PiB PET was used to assess amyloid plaque load in the brain and FDG ‐PET for brain glucose metabolism. FreeSurfer‐based standardized uptake value ratios (fSUVR, with the total cerebellum gray matter as reference region) were corrected for partial volume effects using a regional point spread function. For the assessment of global cortical amyloid burden (cortical PiB PET) and global cortical brain metabolism (cortical FDG PET), the mean SUVR of the precuneus, prefrontal cortex, gyrus rectus, and lateral temporal regions was used. The cut‐off value for the classification of PiB PET amyloid positivity was 1.42. PiB PET was available for 147 and FDG PET for 154 participants.

Regional brain volume and cortical thickness were assessed by MRI. Regional volumes were corrected for head size by normalization to the intracranial volume (ICV) as suggested by the DIAN Imaging Core (Normalized volume = regionalVol – [B‐weight ∗ (individual ICV − mean ICV)]). ^12^ MRI data were available for 156 participants.

### 2.6 Statistics

Statistical analysis was performed using GraphPad Prism v8.3.0 and SPSS v29.0.0.0.

Normal distribution was tested using the Shapiro–Wilk test. Sex distribution and apolipoprotein E (*APOE*) ε4 positivity were compared with the chi‐square test. We used the Kruskal–Wallis test followed by Dunn post hoc test for group comparisons of age, education, and clinical scores. Imaging and fluid biomarker data were log10‐transformed (if not normally distributed) and groups were compared with univariate and multivariate general linear regression including age, sex, education, and *APOE* ε4 status as covariates and Sidak adjustment for multiple testing. Correlation analyses were performed using Spearman rank correlation coefficient including age and sex as covariates. Imaging data were visualized using BrainPainter software.^13^

For longitudinal estimates, serum β‐synuclein levels and other biomarkers at baseline in NC and MC (aMC+sMC) were plotted against the DIAN EYO at baseline. The effects of DIAN EYO and mutation group on serum β‐synuclein levels were calculated using a linear mixed model including family affiliation as a random effect. For each marker and group (NC and carrier), the best regression model (linear, quadratic, cubic, exponential) was selected based on *R*‐squared. The time of change for each biomarker was defined as the point at which the 95% confidence intervals of the regression lines for NC and MC did not overlap anymore. For visualization and comparison of the longitudinal trajectories of fluid, imaging, and clinical biomarkers in MC relative to the NC group, a *z* score was calculated for each biomarker using mean and standard deviation of the NC group (standardized difference) and plotted using smoothing splines. We performed receiver operating characteristics (ROC) curve analysis to define cortical hypometabolism (based on cortical FDG PET) and hippocampal atrophy and to compare biomarkers for predicting Aβ PiB‐PET positivity, cortical hypometabolism, hippocampal atrophy, and cognitive impairment. Biomarker combinations were tested using binary logistic regression. We used linear regression models to estimate the predictive value of baseline biomarker levels (serum β‐synuclein, CSF pT217/T217) on future cognitive decline. Biomarker levels were used as the predictors and the annual rate of change of MMSE or CDR‐SB as the outcome. Age, sex, and *APOE* ε4 status were included as covariates.

## 3 RESULTS

### 3.1 Participants

We investigated 178 participants including 69 cognitively unimpaired NC, 78 aMC, and 31 sMC. Follow‐up data were available for 49 participants (NC = 16, aMC = 20, sMC = 13). Demographic and clinical characteristics are given in Table 1. To optimally characterize changes in the preclinical AD phase, the NC group (median 38.4 years, interquartile range [IQR] 32.3–47.0) was age‐matched to the aMC group (35.4 years, IQR 30.2–41.9, *p* = 0.13). The sMC group (46.6 years, IQR 42.1–52.6) was older than the NC (*p* = 0.001) and aMC groups (*p *< 0.0001). Sex distribution (*p* = 0.16) and *APOE* ε4 positivity (*p* = 0.80) were not significantly different between the groups. The level of education and cognitive scores were lower in the sMC group compared to aMC (education *p* = 0.003; MMSE and CDR‐SB *p *< 0.0001) and NC (education *p* = 0.0007; MMSE and CDR‐SB *p *< 0.0001).

### 3.2 Serum β‐synuclein levels increase during the preclinical phase

We investigated serum β‐synuclein levels in aMC subjects to study synaptic degeneration in the preclinical phase of ADAD compared to NC and sMC participants. Serum β‐synuclein levels were not associated with age in NC (*r*_s_ = −0.04, *p* = 0.75). Women in the NC group (5.81 pg/mL, IQR 4.78–13.8) had slightly higher serum β‐synuclein levels than men (4.85 pg/mL, IQR 4.00–5.34, *p* = 0.014). There was no difference between *APOE* ε4 allele carriers and non‐carriers (4.90 pg/mL, IQR 3.91–6.57 vs. 5.51 pg/mL, IQR 4.66–6.87, *p* = 0.50).

**FIGURE 1:**
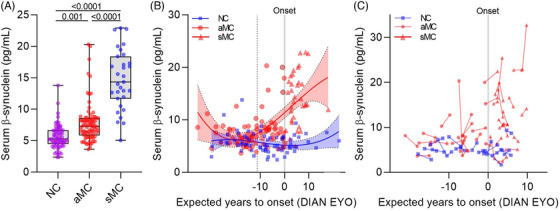
Serum β‐synuclein levels start to increase during the preclinical phase. A, Serum β‐synuclein levels are higher in asymptomatic (aMC, *n* = 78) and symptomatic mutation carriers (sMC, *n* = 31) compared to mutation non‐carriers (NC, *n* = 69) and in sMC versus aMC. Groups were compared by multivariate general linear regression using log10‐transformed β‐synuclein levels, including age, sex, education, and *APOE* ε4 positivity as covariates and Sidak adjustment for multiple comparisons. Boxes are median and interquartile range, whiskers are min and max. Dots are individual values. B, Scatter plot of serum β‐synuclein levels at baseline and expected years to symptom onset (DIAN EYO) in NC (*n* = 69) and MC (*n* = 109) subjects. Data were fitted using non‐linear regression and the start of β‐synuclein increase (11.2 years before onset, indicated by the dotted line) was defined at the point where the 95% confidence intervals of the regression lines diverged. Dots are individual values. The black‐rimmed dots are subjects who converted from the asymptomatic to the symptomatic state during follow‐up. C, Longitudinal changes of serum β‐synuclein levels in individual subjects during follow‐up visits are shown in NC (*n* = 16) and MC (*n* = 33) by connecting lines. B,C, According to the DIAN policy, X axis labeling is limited to +10 to –10 to limit genetic unblinding. APOE, apolipoprotein E; DIAN, Dominantly Inherited Alzheimer's Network.

Serum β‐synuclein levels were higher in aMC compared to NC subjects (*p* = 0.001, Figure 1A) indicating that synaptic degeneration begins early in ADAD. Structural and metabolic changes in AD‐typical brain regions (measured by MRI and FDG PET, Table 1) and axonal neurodegeneration (measured by NfL, Table 1) were not significantly changed in aMC subjects implying that synaptic changes begin earlier. Higher β‐synuclein levels were also observed in sMC compared to NC (*p *< 0.0001) and aMC (*p *< 0.0001).

### 3.3 Longitudinal estimates for serum β‐synuclein and other biomarker changes

We plotted serum β‐synuclein levels in NC and MC participants against DIAN EYO to estimate the longitudinal trajectories of β‐synuclein in both groups (Figure 1B,C). The DIAN EYO is a proxy to estimate years to onset considering the type of mutation and parental age of symptomatic onset. The association between serum β‐synuclein levels and DIAN EYO differed between NC and MC (*p *< 0.001). β‐synuclein levels were constant in NC but increased over time in MC, beginning ≈ 11 years before EYO (Figure 1B). Two subjects in our cohort converted from asymptomatic to symptomatic AD during follow‐up and presented with elevated serum β‐synuclein levels at baseline (Figure 1B,C).

**FIGURE 2:**
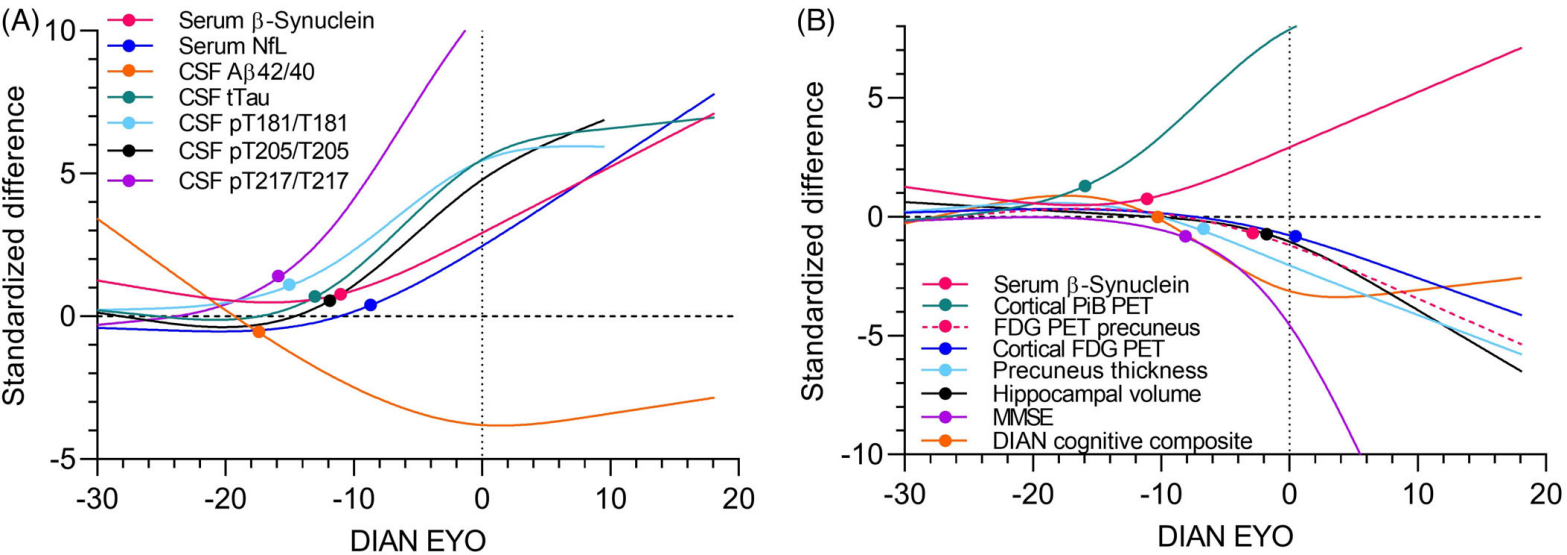
Longitudinal trajectories of biomarkers based on expected years to symptom onset. Values of (A) fluid, (B) imaging, and clinical biomarkers of all subjects were normalized to the NC group by calculating a *z* score using the mean and SD of the NC group (standardized difference). The standardized difference of aMC and sMC subjects are plotted against the expected years to symptom onset (DIAN EYO) and the trajectories are visualized by smoothing splines. The colored dots indicate the point where biomarkers in MC start to differ from the NC group as defined by divergence of the 95% confidence interval of the regression lines. The dotted line represents symptom onset. Aβ, amyloid beta; aMC, asymptomatic mutation carriers; CSF, cerebrospinal fluid; DIAN, Dominantly Inherited Alzheimer's Network; FDG, fluorodeoxyglucose; MMSE, Mini‐Mental State Examination; NC, mutation non‐carriers; NfL, neurofilament light chain; PET, positron emission tomography; PiB, Pittsburgh compound B; pT, phosphorylated tau; SD, standard deviation; sMC, symptomatic mutation carriers; tTau, total tau.

We compared the DIAN EYO–based longitudinal trajectories of serum β‐synuclein levels to other fluids (Figure 2A), imaging, and clinical biomarkers (Figure 2B). Markers of amyloid pathology change first (CSF Aβ42/40: –17.7 years; PiB PET: –16.2 years) followed by p‐tau species (pT217/T217: −16.1 years; pT181/T181: −15.3 years; pT205/T205: −12.1 years) and t‐tau in CSF (−13.0 years). This is followed by serum β‐synuclein (−11.3 years) thereby preceding the axonal degeneration marker NfL (−8.9 years) as well as cognitive decline (cognitive composite: −10.3 years; MMSE: −8.1 years), brain atrophy (precuneus thickness: −6.9 years; hippocampal volume: −1.8 years), and brain glucose metabolism (FDG PET precuneus: −3.0 years; FDG PET cortex: +0.2 years).

### 3.4 Association of serum β‐synuclein levels with amyloid plaque load

**FIGURE 3:**
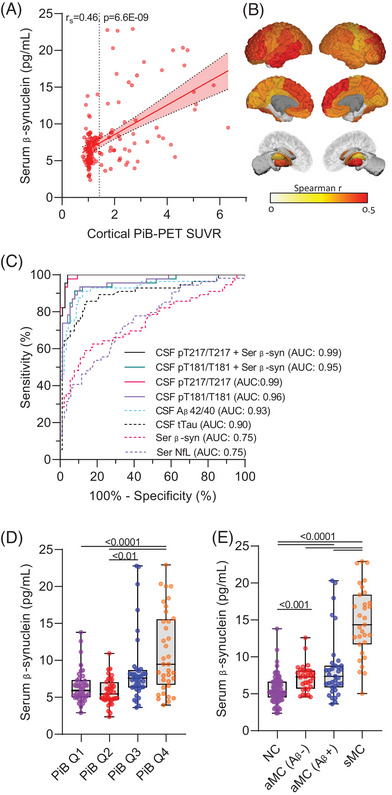
Association of serum β‐synuclein levels with brain amyloid plaque load. There was a significant correlation of serum β‐synuclein levels with (A) cortical PiB PET SUVR and (B) PiB PET SUVR in different cortical and subcortical regions at baseline in the whole cohort. The scatter plot in (A) shows individual values, the dotted line is the defined threshold (1.42) for amyloid positivity, and the red line is a linear regression line including the 95% confidence interval. B, The color code shows the Spearman correlation coefficient (*r*_s_) of brain regions with a significant correlation between serum β‐synuclein and PiB PET SUVR. Correlation analysis in (A) and (B) was performed by partial Spearman correlation including age and sex as covariates and Bonferroni correction for multiple testing. C, Receiver operating characteristic (ROC) curve analysis of serum β‐synuclein levels (Ser β‐syn) and other fluid biomarkers for diagnosing PiB PET–based amyloid positivity in the whole cohort. Biomarker combinations were tested using binary logistic regression. D, Comparison of serum β‐synuclein levels in all subjects (*n* = 147) divided into PiB PET SUVR quartiles (Q1–Q4) showed the first significant changes of β‐synuclein in Q3 (PiB PET SUVR of 1.15–2.12), which also included the cut‐off defining Aβ PiB PET positivity (1.42). E, Comparison of serum β‐synuclein levels in NC, aMC, and sMC subjects to aMC subjects divided into PiB PET amyloid positive (Aβ+) and negative (Aβ−) subjects. Statistical analysis in (D) and (E) was performed by univariate general linear regression including age, sex, education and *APOE* ε4 status as covariates and Sidak adjustment for multiple testing. *p* values are indicated in the graph. Boxes are median and interquartile ranges, whiskers are minimum and maximum, and dots are individual values. Aβ, amyloid beta; aMC, asymptomatic mutation carriers; *APOE*, apolipoprotein E; AUC, area under the curve; NC, mutation non‐carriers; NfL, neurofilament light chain; PiB PET, Pittsburgh compound B positron emission tomography; pT, phosphorylated tau; sMC, symptomatic mutation carriers; SUVR, standardized uptake value ratio; tTau, total tau.

We observed a significant correlation of serum β‐synuclein levels with global cortical amyloid plaque load measured by PiB PET (*r*_s_ = 0.46, Figure 3A). After separation into different cortical and subcortical brain regions, most areas showed a significant correlation with serum β‐synuclein without a clear regional pattern (Figure 3B, Table S1 in supporting information). Diagnostic performance of serum β‐synuclein for (PiB PET–based) amyloid positivity was moderate (area under the curve [AUC] 0.75, 95% confidence interval [CI] 0.66–0.83) and similar to serum NfL (AUC 0.75, 95% CI 0.67–0.84) but considerably lower than CSF markers of amyloid pathology including Aβ42/40 and p‐tau variants (AUC ≥ 0.93, Figure 3C). Combination of CSF pT181/T181 and pT217/T217 with serum β‐synuclein by logistic regression did not improve the already high diagnostic performance of these markers for amyloid positivity (pT181/T181: AUC 0.96, 95% CI 0.92–0.99 vs. AUC 0.95, 95% CI 0.91–0.99; pT217/T217: AUC 0.99, 95% CI 0.98–1.00 vs. AUC 0.99, 95% CI 0.99–1.00, Figure 3C). We divided subjects with available PiB PET into quartiles to investigate the association of serum β‐synuclein levels with amyloid plaque load at different disease stages (Figure 3D). Higher β‐synuclein levels were observed in the third (Q3, 1.15–2.12) and fourth (Q4, 2.12–6.31) quartile of PiB PET SUVRs whereas the cut‐off defining Aβ PiB‐PET positivity (1.42) was within Q3. Separation of aMC into Aβ+ and Aβ− participants revealed significantly higher serum β‐synuclein levels already in Aβ− aMC compared to NC (Figure 3E), further supporting a very early start of synaptic degeneration in AD.

### 3.5 Association of serum β‐synuclein with brain metabolism and atrophy

**FIGURE 4:**
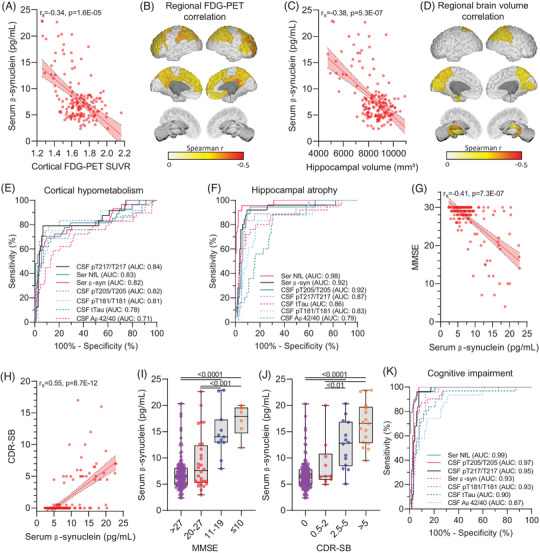
Association of serum β‐synuclein levels with brain volume, brain metabolism, and cognition. There was a significant correlation of serum β‐synuclein levels with brain metabolism (FDG PET) and brain volume (MRI). A, Correlation of serum β‐synuclein with cortical FDG PET SUVR and (B) FDG PET SUVR in different cortical and subcortical regions at bassline in the whole cohort. C, Correlation of serum β‐synuclein with hippocampal volume and (D) MRI‐derived normalized volume in different cortical and subcortical regions at baseline in the whole cohort. The scatter plots in (A,C) show individual values and the red line is a linear regression line including the 95% confidence interval. The color code in (B,D) shows the Spearman correlation coefficient (*r*_s_) of brain regions with a significant correlation between serum β‐synuclein and FDG PET SUVR or MRI‐derived cortical/subcortical volume. Correlation analysis in (A‐D) was performed by partial Spearman correlation including age and sex as covariates and Bonferroni correction for multiple testing. E,F, ROC analysis for the prediction of (E) cortical hypometabolism defined as a cortical FDG PET SUVR < 1.537 and (F) hippocampal atrophy defined as hippocampal volume < 7542mm^3^. G,H, Spearman correlation analysis of serum β‐synuclein levels with the (G) MMSE and (H) CDR‐SB scores. The scatter plots show individual values and the red line is a linear regression line including the 95% confidence interval. I,J, Subjects were grouped according to their cognitive performance using the (I) MMSE and (J) CDR‐SB. Serum β‐synuclein levels in MMSE/CDR‐SB groups were compared by univariate general linear regression including age, sex, education, and *APOE* ε4 status as covariates and Sidak adjustment for multiple testing. *p* values are indicated in the graph. Boxes are median and interquartile ranges, whiskers are minimum, and maximum and dots are individual values. K, ROC analysis comparing the diagnostic performance of fluid biomarkers to separate cognitively unimpaired participants (global CDR 0) from those with cognitive impairment (global CDR > 0). Aβ, amyloid beta; *APOE*, apolipoprotein E; AUC, area under the curve; CDR‐SB, Clinical Dementia Rating Sum of Boxes; CSF, cerebrospinal fluid; FDG PET, fluorodeoxyglucose positron emission tomography; MMSE, Mini‐Mental State Examination; MRI, magnetic resonance imaging; NfL, neurofilament light chain; pT181/T181, pT205/T205, pT217/T217, ratio of phosphorylated and unphosphorylated tau protein at positions 181, 205, and 217; ROC, receiver operating characteristic curve; Ser, serum; SUVR, standardized uptake value ratio; tTau, total tau protein.

Serum β‐synuclein levels correlated with global cortical brain metabolism as measured by FDG PET (*r*_s_ = –0.34, Figure 4A). On a regional scale, there was a moderate correlation with some frontal and parietal areas (Figure 4B, Table S1) including the precuneus but not in subcortical regions. We observed a significant correlation of serum β‐synuclein with hippocampal volume (*r*_s_ = –0.38, Figure 4C) and with other temporal (entorhinal cortex), parietal (inferiorparietal, superparietal and supramarginal lobe, precuneus), occipital (cuneus, lingual gyrus) and subcortical (thalamus, accumbens, putamen) structures (Figure 4D, Table S1). We used receiver operating characteristic (ROC) curve analysis of cortical FDG PET SUVR and hippocampal volume in NC and sMC to define cutoffs for cortical hypometabolism (FDG ‐PET SUVR  < 1.537) and hippocampal atrophy (hippocampal volume  < 7542mm^3^) using the Youden index. Serum β‐synuclein showed a moderate performance to predict hypometabolism (AUC 0.82) whereas serum NfL (AUC 0.83) and CSF pT217/T217 (AUC 0.84) performed slightly better (Figure 4E). For the prediction of hippocampal atrophy, serum β‐synuclein showed good performance (AUC 0.92) and only serum NfL performed better (AUC 0.98, Figure 4F).

### 3.6 Association of serum β‐synuclein with fluid biomarkers and cognitive impairment

There was a strong correlation of serum β‐synuclein levels with the other fluid biomarkers including markers for axonal and general neurodegeneration (serum NfL, CSF t‐tau) and amyloid pathology (CSF Aβ42/40 and p‐tau species) except for pT202/T202 (*r*_s_ = –0.32; Table 2). Strongest correlation was observed with CSF pT217/T217 (*r*_s_ = 0.62) and serum NfL (*r*_s_ = 0.60). A significant moderate‐to‐strong correlation of serum β‐synuclein was also observed with cognitive function (CDR‐SB, MMSE, DIAN cognitive composite) with the strongest association with the CDR‐SB (*r*_s_ = 0.55, Table 2, Figure 4G,H). We divided the cohort into groups with different degrees of cognitive impairment to see how serum β‐synuclein levels reflect different stages of AD (Figure 4I,J). Grouping was done with two different scores, MMSE and CDR‐SB. A similar picture was observed in both cases with a rise of serum β‐synuclein levels with increasing decline in cognition (Figure 4I,J). Serum β‐synuclein showed a good performance in separating cognitively unimpaired participants from those with cognitive impairment (AUC 0.93, Figure 4K) whereas serum NfL (AUC 0.99) was the best‐performing fluid biomarker here.

**TABLE 2:** Correlation of fluid biomarkers and cognitive scores at baseline.

	Serum NfL	CSF Aβ42/40 ratio	CSF tTau	CSF pT181/T181	CSF pT205/T205	CSF pT202/T202	CSF pT217/T217	CDR‐SB	MMSE	DIAN cognitive composite
Serum β‐synuclein	0.60^****^	−0.56^****^	0.50^****^	0.59^****^	0.54^****^	−0.32^***^	0.62^****^	0.55^****^	−0.41^****^	−0.39^****^
Serum NfL		−0.44^****^	0.41^****^	0.49^****^	0.52^****^	−0.15	0.48^****^	0.60^****^	−0.41^****^	−0.40^****^
CSF Aβ42/40 ratio			−0.61^****^	−0.71^****^	−0.54^****^	0.27^**^	−0.75^****^	−0.44^****^	0.35^****^	0.32^***^
CSF tTau				0.65^****^	0.51^****^	−0.56^****^	0.68^****^	0.47^****^	−0.45^****^	−0.36^****^
CSF pT181/T181					0.60^****^	−0.33^***^	0.76^****^	0.53^****^	−0.40^****^	−0.35^****^
CSF pT205/T205						0.04	0.60^****^	0.55^****^	−0.45^****^	−0.42^****^
CSF pT202/T202							−0.44^****^	−0.14	0.23^**^	0.23^**^
CSF pT217/T217								0.58^****^	−0.47^****^	−0.41^****^
CDR‐SB									−0.64^****^	−0.52^****^
MMSE										0.67^****^

*Note*: Values are Spearman correlation coefficients from partial Spearman correlation analysis including age and sex as covariates. Only individuals without missing values for any of the variables were included (*n* = 135).

Abbreviations: Aβ, amyloid beta; *APOE*, apolipoprotein E; CDR‐SB, Clinical Dementia Rating Sum of Boxes; CSF, cerebrospinal fluid; DIAN, Dominantly Inherited Alzheimer's Network; FDG, fluorodeoxyglucose; MMSE, Mini‐Mental State Examination; NfL, neurofilament light chain; PET, positron emission tomography; PiB, Pittsburgh compound B; pT, phosphorylated tau; SUVR, standardized uptake value ratio; tTau, total tau protein.

** *p *< 0.01

*** *p *< 0.001

**** *p *< 0.0001

Follow‐up data were available for a small subset of participants only. In an exploratory approach, we estimated the predictive value of baseline serum β‐synuclein levels for future cognitive decline in MC measured by the annual rate of change of the MMSE (*n* = 24) and CDR‐SB (*n* = 25) scores (mean follow‐up time 1.89 ± 0.95 years). Serum β‐synuclein showed a significantly better prediction (MMSE: *R* ^2^ = 0.50, *p *< 0.01; CDR‐SB: *R* ^2^ = 0.54, P*p* = 0.001) than the covariates‐only model (i.e., age, sex, *APOE* ε4 status; MMSE: *R* ^2^ = 0.17; CDR‐SB: *R* ^2^ = 0.17) and slightly better than CSF pT217/T217 (MMSE: *R* ^2^ = 0.43; CDR‐SB: *R* ^2^ = 0.42). Addition of serum β‐synuclein to the model with CSF pT217/T217 significantly improved prediction of future cognitive decline (MMSE: *R* ^2^ = 0.55, *p* = 0.046; CDR‐SB: *R* ^2^ = 0.56, *p* = 0.02).

## 4 DISCUSSION

We here showed that serum β‐synuclein levels are already raised in aMC and are highest in sMC. Longitudinal estimations suggest that β‐synuclein levels become abnormal 11 years before symptom onset, representing an early marker of AD pathophysiology, changing after amyloid‐related markers became positive, and preceding changes in brain structure (i.e., atrophy) and metabolism (i.e., FDG PET), cognition, and axonal neurodegeneration. These findings suggest that synaptic degeneration is one of the earliest events in ADAD pathophysiology and is indicative of a retrograde degeneration mechanism starting in the synapse.

The aim of our study was to characterize the changes in serum β‐synuclein levels, an easily accessible marker of synaptic degeneration, in the asymptomatic phase of ADAD and estimate the longitudinal trajectories in relation to other disease mechanisms. Our data showed that serum β‐synuclein rises very early in ADAD indicated by higher levels in aMC compared to controls. We observed higher β‐synuclein levels even in aMC subjects that had not reached the PiB PET cut‐off for amyloid positivity indicating that synaptic degeneration belongs to the earliest events in AD after the start of amyloid deposition. This is further supported by higher β‐synuclein levels first observed in PiB PET quartile Q3, including the cut‐off for amyloid positivity, and by the estimated biomarker trajectories based on the DIAN EYO. We could show that serum β‐synuclein levels start to increase ≈ 11 years before symptom onset. Thus, it is the first marker to rise (other than CSF t‐tau) after the amyloid‐related markers (PiB PET, CSF Aβ42/40, CSF p‐tau variants) and preceding axonal degeneration (serum NfL), cognitive decline, brain atrophy (MRI), and metabolism (FDG PET). In agreement with this, other studies in DIAN have reported a very early increase of synaptic markers in CSF of ADAD.^14^, ^15^ Based on its strong correlation with other synaptic markers in CSF including CSF β‐synuclein,^4^ CSF t‐tau has recently been discussed to better reflect synaptic degeneration than general neurodegeneration as previously thought.^16^ This would also better integrate into the observed temporal order of biomarkers and further support our hypothesis of the very early start of synaptic damage in AD. Serum β‐synuclein levels are also more strongly correlated with amyloid pathology (PiB PET) than with brain atrophy and metabolism, which further supports a tighter temporal proximity of synaptic degeneration to amyloid deposition. On the other hand, it is unlikely that serum β‐synuclein levels reflect just amyloid pathology because it showed only moderate performance to diagnose amyloid positivity and did not have an added value to the established markers pT217 and pT181. Thus, there are several pieces of evidence from the present and previous studies ranking synaptic degeneration to the earliest events in ADAD pathophysiology which can be detected by serum β‐synuclein measurement.

The estimated temporal order of biomarker changes in our study is very similar to previous reports from ADAD^8^, ^17^ although the estimated number of years when changes start to appear are not identical. We ascribe this to different statistical models used to define the time of change but the fact that different models provide a similar temporal order supports the robustness of these findings. Also, the change of cognitive decline (cognitive composite and MMSE) is ranked earlier in our study compared to previous DIAN studies^14^ which might result from the inclusion of different DIAN participants and also slightly lower sample size in our study compared to previous ones.^8^, ^14^ Within our study, we could add a marker of synaptic degeneration to this puzzle and temporally associate it with early amyloid deposition. Serum β‐synuclein levels also changed before the axonal marker NfL. It is therefore tempting to speculate that the degeneration process starts at the synapse and retrogradely progresses through the axon to the neuronal soma, although a marker for the latter is lacking so far in this cascade and must be confirmed. The synapse has been proposed as the initial site of neurodegeneration for many years and there is also evidence from other studies that it precedes axonal degeneration.^15^, ^18^, ^19^ Our data show that we are now able to track this processes with easily accessible blood markers in human subjects.

Our data are consistent with our previous observation in Down syndrome (DS) subjects.^20^ DS, that is, trisomy 21, is characterized by triplication of the *APP* gene and represents a genetic form of AD. Here, we showed higher serum β‐synuclein levels in asymptomatic DS subjects without clinical signs of AD and highest levels in DS with AD, similar to our study in ADAD. Our data in ADAD are also in line with our studies in sporadic AD showing a gradual increase of β‐synuclein levels from Aβ+ but cognitively normal subjects, mild cognitive impairment AD, and AD dementia^5^, ^6^, ^7^ indicating that the temporal estimations from ADAD are transferable to sporadic AD. Other studies also support that fluid biomarker profiles are similar in ADAD and sporadic AD whereas longitudinal clinical presentation might vary^21^ but this must be confirmed for β‐synuclein.

Synaptic degeneration is the pathological correlate of memory impairment in AD^22^ and synaptic markers might be used as surrogate markers to track or predict changes of cognition. We here showed a significant correlation of serum β‐synuclein levels with three different measures of cognitive function (MMSE, CDR‐SB, DIAN cognitive composite) in agreement with our previous observations in sporadic AD.^5^, ^7^ We divided the participants according to their degree of impairment using the MMSE and CDR‐SB scores. With both scores, β‐synuclein levels strongly depended on the degree of cognitive impairment suggesting a gradual increase with declining cognitive function. Furthermore, the ROC curve analysis identified serum β‐synuclein as a good predictor of cognitive impairment. These data support the use of serum β‐synuclein as a surrogate biomarker for cognitive changes. Our exploratory data, indicating a predictive value of β‐synuclein for future cognitive decline in combination with CSF pT217/T217, are also promising but need further verification due to the small sample size.

Clinical scores are an important tool in AD drug development and usually the main outcome measure in clinical trials to show the efficacy of a drug. The recent success of anti‐amyloid drugs was demonstrated in patients with early symptomatic AD by a slower increase of the CDR‐SB score^3^ and greater time to progression to the next stage of dementia.^23^ Surrogate markers of clinically meaningful disease processes are urgently needed to support the design and operationalization of clinical trials of putative AD‐modifying therapies at earlier stages of disease—including in asymptomatic (cognitively normal) participants in whom clinical read‐outs are impractical. Synaptic markers are promising candidates here and the estimated trajectories from our study suggest that synaptic markers such as β‐synuclein might be helpful in two ways. According to our data, synaptic degeneration is one of the earliest events in the preclinical phase after start of amyloid deposition. The monitoring of synaptic markers in amyloid‐positive subjects will indicate the time when synaptic degeneration starts. This could be an ideal point to start anti‐amyloid therapy thereby also excluding the substantial number of subjects with benign amyloid deposition that will never develop AD during their lifetime. Monitoring of synaptic markers during treatment might be an important read‐out to evaluate positive treatment effects in conjunction with amyloid‐related markers for target engagement because patients are still cognitively normal. Data from anti‐amyloid trials^3^ also support the use of synaptic markers to monitor treatment effects. Serum β‐synuclein is a promising candidate here because it is easily accessible also for longitudinal follow‐up. In addition, it is less dependent on age compared to other blood biomarkers (e.g., NfL, GFAP) which is advantageous for longitudinal monitoring. However, data from anti‐amyloid trials are only available for CSF synaptic markers so far and correlation of CSF and blood biomarkers can vary strongly. One contributing factor here can be peripheral expression of markers interfering with brain‐derived changes, which has been shown, for example, for neurogranin.^24^ Studies to evaluate blood β‐synuclein as a read‐out in clinical trials are therefore urgently needed.

A limitation of our study could be the sample size, which is smaller than in previous DIAN studies and might explain some differences in the longitudinal estimates. However, the group differences of serum β‐synuclein are highly significant and do not require a larger sample size to increase statistical power. Cross‐sectional differences of other variables are also similar to previous DIAN studies supporting a similar composition of our cohort and comparability. We had follow‐up samples only for a subset of participants to calculate the rate of change for serum β‐synuclein which might be a better measure of longitudinal changes as suggested for other biomarkers in DIAN studies.^25^ The investigation of serum β‐synuclein rate of change should be addressed in more detail in subsequent studies. Also, data on blood p‐tau217, the currently most promising AD blood biomarker, were not yet available in the DIAN data freeze 15 used in the present study and precluded a direct comparison to blood β‐synuclein which would be important to better estimate the added value of β‐synuclein and requires further studies.

In conclusion, our data indicate that synaptic degeneration starts early in asymptomatic ADAD. It is ranked as one of the first events after start of amyloid deposition and preceding axonal degeneration, brain atrophy, and metabolism in support of a retrograde degenerative process starting in the synapse. Serum β‐synuclein is an easily accessible biomarker to track synaptic changes and is associated with cognitive impairment and might be used in drug trials for patient selection and monitoring of treatment effects. Longitudinal studies are needed to confirm our temporal estimates and determine that findings in ADAD are generalizable to sporadic AD.

## AUTHOR CONTRIBUTIONS

Conception and design of the work: Patrick Oeckl, Markus Otto. Acquisition, analysis, or interpretation of data: Patrick Oeckl, Benjamin Mayer, Randall J. Bateman, Gregory S. Day, Nick C. Fox, Edward D. Huey, Laura Ibanez, Takeshi Ikeuchi, Mathias Jucker, Jae‐Hong Lee, Johannes Levin, Jorge J. Llibre‐Guerra, Francisco Lopera, Eric McDade, John C. Morris, Yoshiki Niimi, Jee Hoon Roh, Raquel Sánchez‐Valle, Peter R. Schofield, Markus Otto. Drafting of the manuscript: Patrick Oeckl, Markus Otto. All authors critically revised the work for important intellectual content, finally approved the version to be published, and agreed to be accountable for all aspects of the work in ensuring that questions related to the accuracy or integrity of any part of the work are appropriately investigated and resolved.

## CONFLICT OF INTEREST STATEMENT

Patrick Oeckl received research support from the Alzheimer Forschung Initiative e.V. (20059CB), ALS Association/ALS Finding A Cure (24‐SGP‐691, 23‐PPG‐674‐2), Charcot Foundation (D.7090), DZNE Innovation‐to‐Application (I2A_call7_Oeckl, I2A_call9_Oeckl), consulting fees from LifeArc and Fundamental Pharma, and travel support from Biogen. Markus Otto received research support from German Federal Ministry of Education and Research (FTLDc 01GI1007A), the EU Joint Programme‐Neurodegenerative Diseases networks Genfi‐Prox (01ED2008A), the EU (MOODMARKER 01EW2008), the German Research Foundation/DFG (SFB1279), the foundation of the state Baden‐Württemberg (D.3830), Boehringer Ingelheim Ulm University BioCenter (D.5009), and the Thierry Latran Foundation. Markus Otto and Patrick Oeckl are co‐inventors of a patent application for using β‐synuclein measurement in blood (EP4014048A1, US2022283184A1). Johannes Levin reports speaker fees from Bayer Vital, Biogen, EISAI, TEVA, Esteve, Zambon, and Roche; consulting fees from Axon Neuroscience, EISAI, and Biogen; author fees from Thieme medical publishers and W. Kohlhammer GmbH medical publishers; and is inventor in a patent “Oral Phenylbutyrate for Treatment of Human 4‐Repeat Tauopathies” (EP 23 156 122.6) filed by LMU Munich. In addition, he reports compensation for serving as chief medical officer for MODAG GmbH, is beneficiary of the phantom share program of MODAG GmbH, and is inventor in a patent “Pharmaceutical Composition and Methods of Use” (EP 22 159 408.8) filed by MODAG GmbH, all activities outside the submitted work. Jorge J. Llibre‐Guerra's research is supported by NIH‐NIA (K01AG073526), the Alzheimer's Association (AARFD‐21‐851415, SG‐20‐690363), the Michael J. Fox Foundation (MJFF‐020770), the Foundation for Barnes‐Jewish Hospital, and the McDonnell Academy. Francisco Lopera has grants and support from NIA, NIH, RED‐LAT, LATAM‐Fingers, Large PD, Roche, Biogen, Tau Consortium, Alzheimer Association, and Viewmind. All other authors declare no competing interests. Author disclosures are available in the supporting information.

## CONSENT STATEMENT

All participants gave written informed consent to participate in the study.

## COLLABORATORS OF THE DOMINANTLY INHERITED ALZHEIMER NETWORK

David Aguillon, Ricardo F. Allegri, Andrew J. Aschenbrenner, Bryce Baker, Nicolas Barthelemy, Randall Bateman, Jacob A. Bechara, Tammie Benzinger, Sarah B. Berman, William S. Brooks, David M. Cash, Allison Chen, Charles Chen, Jasmeer P. Chhatwal, Patricio Chrem Mendez, Laura Courtney, Carlos Cruchaga, Alisha J. Daniels, Gregory Day, Anne M. Fagan, Martin Farlow, Shaney Flores, Nick C. Fox, Erin Franklin, Alison M. Goate, Brian A. Gordon, Susanne Graber‐Sultan, Neill R. Graff‐Radford, Emily Gremminger, Jason Hassenstab, Elizabeth Herries, Anna Hofmann, David M. Holtzman, Russ Hornbeck, Edward D. Huey, Laura Ibanez, Takeshi Ikeuchi, Snezana Ikonomovic, Kelley Jackson, Steve Jarman, Gina Jerome, Erik C.B Johnson, Nelly Joseph‐Mathurin, Mathias Jucker, Celeste M. Karch, Kensaku Kasuga, Sarah Keefe, Deborah Koudelis, Elke Kuder‐Buletta, Christoph Laske, Jae‐Hong Lee, Yudy Milena Leon, Allan I. Levey, Johannes Levin, Yan Li, Jorge J. Llibre‐Guerra, Francisco Lopera, Ruijin Lu, Jacob Marsh, Ralph Martins, Parinaz Massoumzadeh, Colin Masters, Austin McCullough, Eric McDade, Nicole McKay, Matthew Minton, Hiroshi Mori, John C. Morris, Neelesh K. Nadkarni, Joyce Nicklaus, Yoshiki Niimi, James M. Noble, Ulrike Obermueller, Richard J. Perrin, Danielle M. Picarello, Christine Pulizos, Laura Ramirez, Alan E. Renton, John Ringman, Jacqueline Rizzo, Yvonne Roedenbeck, Jee Hoon Roh, Pedro Rosa‐Neto, Natalie S. Ryan, Edita Sabaredzovic, Stephen Salloway, Raquel Sanchez‐Valle, Peter R. Schofield, Jalen Scott, Nicholas T. Seyfried, Ashlee Simmons, Jennifer Smith, Hunter Smith, Jennifer Stauber, Sarah Stout, Charlene Supnet‐Bell, Ezequiel Surace, Silvia Vazquez, Jonathan Vöglein, Guoqiao Wang, Qing Wang, Chengie Xiong, Xiong Xu, Jinbin Xu.

## Supporting information



Supporting Information

Supporting Information
